# Herpetopanone, a diterpene from *Herpetosiphon aurantiacus* discovered by isotope labeling

**DOI:** 10.3762/bjoc.13.242

**Published:** 2017-11-17

**Authors:** Xinli Pan, Nicole Domin, Sebastian Schieferdecker, Hirokazu Kage, Martin Roth, Markus Nett

**Affiliations:** 1 Department of Biochemical and Chemical Engineering, Technical Biology, Technical University Dortmund, Emil-Figge-Strasse 66, 44227 Dortmund, Germany; 2 Leibniz Institute for Natural Product Research and Infection Biology, Hans Knöll Institute, Beutenbergstr. 11a, 07745 Jena, Germany

**Keywords:** genome mining, herpetopanone, *Herpetosiphon*, isotope labeling, terpene

## Abstract

The genome of the predatory bacterium *Herpetosiphon aurantiacus* 114-95^T^ harbors a number of biosynthesis genes, including four terpene cyclase genes. To identify the terpenes biosynthesized from *H. aurantiacus* 114-95^T^, we fed the strain with ^13^C-labeled glucose and, subsequently, searched for characteristic mass shifts in its metabolome. This approach led to the discovery of a new natural product, of which the isotope pattern is indicative for a diterpene originating from the methylerythritol phosphate pathway. After large-scale fermentation of *H. aurantiacus* 114-95^T^, the putative diterpene was isolated in sufficient quantity to enable NMR-based structure elucidation. The compound, for which the name herpetopanone is proposed, features a rare octahydro-1*H*-indenyl skeleton. Herpetopanone bears resemblance to cadinane-type sesquiterpenes from plants, but is structurally entirely unprecedented in bacteria. Based on its molecular architecture, a possible biosynthetic pathway is postulated.

## Introduction

Terpenoids represent the largest group of natural products with about 60,000 different compounds being known. They occur in all three domains of life and are known to fulfill a variety of different functions, e.g., as membrane constituents, chemical attractants or feeding deterrents [1]. Over a long period, terpenoids were mainly reported from plants and, to a lesser degree, also from fungi and marine invertebrates. In recent years, however, the discovery of terpenoids from prokaryotes has gained momentum. The ease of DNA sequencing has strongly favored this development, contributing to the identification of numerous bacterial terpene cyclase genes [2–3]. Both in vitro and in vivo approaches involving recombinant enzymes are commonly pursued for their functional characterization [4]. Care must be taken, however, in interpreting the results of these analyses, as the products of terpene cyclases are often subject to enzymatic modifications in their native producers. The exclusive testing of a terpene cyclase might hence unveil a biosynthetic intermediate rather than the final product of a secondary metabolite pathway [5–6]. To avoid this problem, we here describe a method for the identification of terpenoids in their natural bacterial hosts, which is based on the feeding of isotopically labeled glucose.

**Figure 1:**
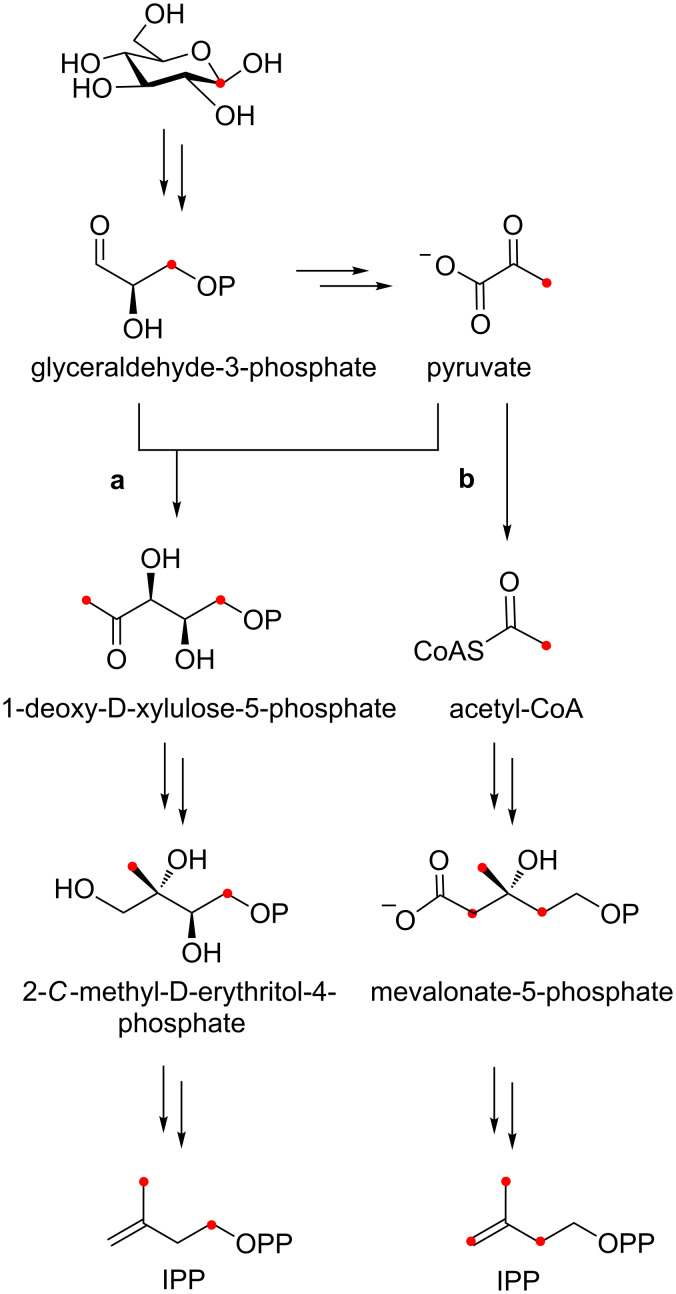
Distribution of isotopic labels from [1-^13^C]-glucose via the MEP (route a) and MEV pathway (route b). Potentially labeled carbon atoms are highlighted with red dots.

The linear oligoprenyl units, which constitute the carbon backbones of terpenoids, arise from the condensation of activated isoprene units, namely isopentenyl diphosphate (IPP) and dimethylallyl diphosphate (DMAPP). The latter two precursors are synthesized by either the mevalonate (MEV) or methylerythritol phosphate (MEP) pathway [7]. Both the MEV and MEP pathway branch from glycolysis. Depending on the respective route, the metabolism of singly labeled glucose gives rise to a characteristic carbon labeling pattern in IPP and DMAPP (Figure 1) [8]. This feature has proven extremely useful to unravel complex cyclization cascades and carbon–carbon rearrangements in the biosynthesis of some terpenoids [9–12]. We anticipated that the resulting mass shifts could also be valuable in the field of natural product discovery. By comparing the ion chromatograms of cultures that were grown in the presence or absence of singly labeled glucose, it might be possible to identify terpenoid natural products in a complex metabolic background.

To validate the feasibility of this approach, we chose the predatory bacterium *Herpetosiphon aurantiacus* 114-95^T^ as a test organism. This strain is capable to produce a variety of polyketides and nonribosomal peptides [13–15] and possesses pathways to supply specific building blocks for these natural products [16]. Furthermore, genomic analyses revealed that *H. aurantiacus* 114-95^T^ features four genes coding for putative terpene cyclases, i.e., Haur_2145, Haur_2987, Haur_2988, and Haur_4149. While the class II cyclase Haur_2145 had already been associated with the production of the terpenoid *O*-methylkolavelool [17], the products of the other three enzymes have not been identified from *H. aurantiacus* 114-95^T^.

## Results and Discussion

**Figure 2:**
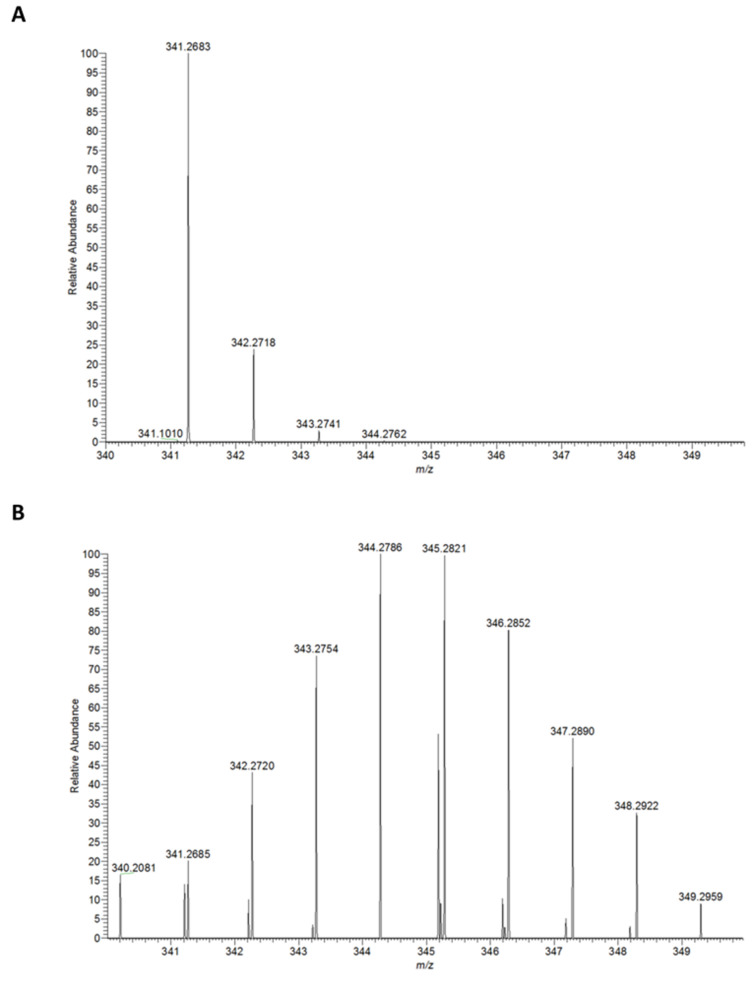
High-resolution mass spectra of a metabolite from *H. aurantiacus* obtained after feeding of unlabeled (A) and [1-^13^C]-labeled D-glucose (B).

For the metabolic labeling experiment, *H. aurantiacus* 114-95^T^ was grown in modified Van Niel’s yeast (VNY) medium. We had previously observed that *Herpetosiphon* cultures grown in this medium develop a deep orange color due to an increased production of carotenoids and it was hence speculated that VNY medium might also support the biosynthesis of other terpenoids. The cultivation was conducted for 7 days in the presence of [1-^13^C]-labeled D-glucose. Cultures supplemented with non-labeled D-glucose served as a control. Extracts from the different *Herpetosiphon* cultures (labeled vs control) were subjected to LCMS analyses. From the comparison of the respective spectra, a possible terpene was identified. The candidate compound lacked absorption maxima in the visible region of the spectrum and could therefore not represent a carotenoid. In our control cultures, the compound exhibited a pseudomolecular ion at *m*/*z* 341.2 [M + H]^+^. After feeding of [1-^13^C]-glucose, however, the same molecule showed a complex isotope pattern with a gradual increase of its *m*/*z* value up to a maximum of 8 Da (Figure 2).

Two conclusions were drawn from this observation. First, it was evident that the isotope labeling was incomplete and that the incorporation of non-labeled metabolic intermediates had led to a mixture of isotopologues and isotopomers. Second, the maximal incorporation of eight labeled carbons was not possible if the compound originated from the MEV pathway (Figure 1). The possible number of labeled carbon atoms was compatible, however, with an octaketide origin or with a diterpene from the MEP pathway. We then recorded the high resolution (HR) mass of the unlabeled compound and determined its molecular formula as C_20_H_36_O_4_. Although the elemental composition does not necessarily exclude a polyketide origin, it perfectly matches a diterpene comprising four intact isoprene units.

To obtain sufficient material for structure elucidation, the fermentation of *H. aurantiacus* 114-95^T^ was repeated on a 50 L scale in VNY medium supplemented with non-labeled D-glucose. The resulting culture broth was centrifuged, and the supernatant was treated with the resin XAD-2 to adsorb the metabolites that had been secreted during the cultivation. After extraction of the adsorber resin with methanol, a preliminary fractionation was accomplished by flash column chromatography on RP silica gel. Fractions containing the target compound were identified by LC–MS, pooled and further purified via semipreparative RP–HPLC to give 19 mg of herpetopanone (**1**).

The IR spectrum of **1** revealed two distinctive bands at 3395 and 1699 cm^−1^, which were assigned to hydroxyl and carbonyl stretching vibrations, respectively. The molecular formula of **1**, which had already been established as C_20_H_36_O_4_, corresponds to three degrees of unsaturation. The signals in the ^13^C NMR spectrum were allocated by DEPT measurements to five methyl, six methylene and six methine groups, as well as three quaternary carbons (Table 1). Only a single carbon (C-19) was sp^2^-hybridized; its resonance at δ 212.2 ppm indicated the presence of a ketone group. It was hence concluded that the structure of **1** must comprise two ring structures in order to comply with the required degrees of unsaturation. Consolidating IR, MS and DEPT data, three hydrogen atoms were assigned to hydroxy groups. The corresponding groups were placed next to C-2, C-4 and C-12 on the basis of ^13^C chemical shifts.

**Table 1:** NMR spectroscopic data of herpetopanone (**1**) in chloroform-*d*_1_.^a^

position	δ_C_ [ppm]	δ_H_ [ppm], M (*J* in Hz)	HMBC

1	23.1, CH_3_	1.14, s	2, 3, 4
2	73.7, C_q_		
3	26.4, CH_3_	1.19, s	1, 2, 4
4	79.0, CH	3.31, dd (9.8, 2.1)	1, 2, 3, 5, 6
5	29.8, CH_2_	a: 1.43, nr b: 1.19, nr	
6	33.1, CH_2_	a: 1.46, nr b: 1.20, nr	5, 8 5, 8
7	34.9, CH	1.19, nr	8
8	13.9, CH_3_	0.68, d (6.2)	6, 7, 9
9	47.6, CH	1.14, nr	8
10	23.2, CH_2_	a: 1.52, nr b: 1.10, nr	9, 11 9, 11
11	41.7, CH_2_	a: 1.79, nr b. 1.36, nr	9, 13, 14 9, 10, 13, 14
12	73.8, C		
13	20.0, CH_3_	1.18, s	11, 12, 14
14	56.7, CH	1.49, nr	13
15	25.2, CH_2_	a: 1.80, nr b: 1.40, nr	12, 14, 18 12, 14
16	28.5, CH_2_	a: 1.94, dddd (13.3, 11.7, 10.0, 8.0) b: 1.56, dddd (13.3, 9.3, 5.5, 1.5)	14, 15, 17, 18, 19 14, 15, 17, 18, 19
17	55.9, CH	2.59, ddd (11.7, 9.5, 5.6)	9, 15, 16, 18, 19, 20
18	46.4, CH	1.81, nr	7, 10, 16
19	212.2, C_q_		
20	29.3, CH_3_	2.16, s	16, 17, 18, 19

^a^nr, not resolved (chemical shift was deduced from the HSQC spectrum).

**Figure 3:**
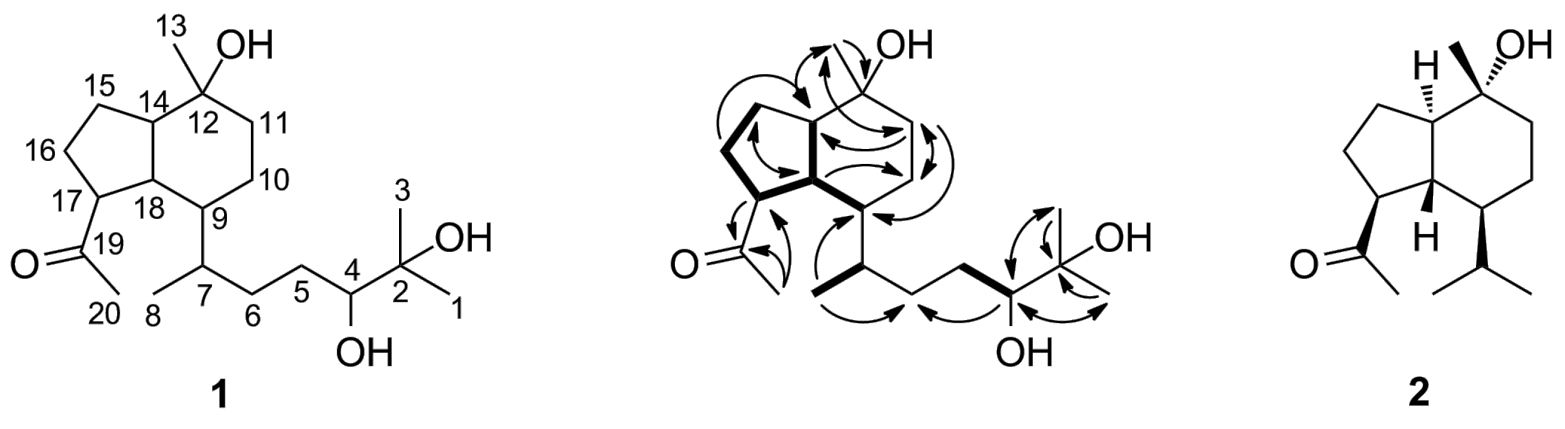
Structures of herpetopanone (**1**) and oplopanone (**2**), as well as selected COSY (bold lines) and HMBC (arrows) interactions in **1**.

While the methyl protons of **1** were readily identified in the ^1^H NMR spectrum, the assignment of several other protons and the readout of their coupling constants was challenging due to severe signal overlapping. A TOCSY spectrum suggested that, with the exception of four methyl groups (CH_3_-1, CH_3_-3, CH_3_-13, CH_3_-20), all protons were part of the same spin system. Structure elucidation started with CH_2_-16 and CH-17, because the protons of these two adjacent groups showed discrete signals in the ^1^H NMR spectrum. Their multiplicities as well as the associated *J* values indicated further vicinal coupling partners. In case of H-16a and H-16b, correlations were also observed with H-15a and H-15b in the COSY spectrum, while H-18 was identified as a coupling partner of H-17. Homonuclear correlations from H-18 to H-9 and H-14 enabled us to expand the deduced partial structure, which was then verified through HMBC and HSQC data. Heteronuclear long-range interactions established the missing substituent at C-17 as an acetyl group and allowed the closure of the cyclopentyl ring in **1** (Figure 3). The proton signal of CH_3_-13 appeared as a singlet, thereby excluding a hydrogen-bearing carbon as an immediate neighbor. In the HMBC spectrum, H_3_-13 showed three heteronuclear correlations, of which only one occurred with a quaternary carbon (C-12). It was hence evident that the methyl group is attached to the hydroxy-substituted C-12, which itself is further connected with C-11 and C-14. HMBC interactions from H-15a and H-15b to C-12 lend additional support for the placement of the latter. The protons of the methylene group in position 11 are magnetically non-equivalent. This feature led to the pairwise occurrence of correlation peaks in the HMBC spectrum and promoted the identification of their ^13^C coupling partners. In addition to the expected interactions with C-13 and C-14, H-11a and H-11b showed correlations to C-9 and C-10. From the observation that H-18 exhibited an HMBC correlation to C-10, but not to C-11, we deduced the octahydro-1*H*-indene scaffold of **1**. The highly shielded protons of CH_3_-8 exhibited HMBC correlations to C-6, C-7, and C-9. Although their vicinal coupling partner could not be unequivocally assigned in the COSY spectrum, the splitting of the corresponding signal was only consistent with a methine being attached to a methyl group. Since CH_3_-8 could not be placed next to CH-9, it had to be linked with CH-7. The two methyl groups, CH_3_-1 and CH_3_-3, appeared as singlets in the ^1^H NMR spectrum. They were positioned next to the quaternary carbon C-2 on the basis of HMBC interactions. Interpretation of 2D NMR data allowed an extension of this residue with CH-4 and CH_2_-5. Ultimately, a heteronuclear long-range correlation from H-4 to C-6 linked the two fragments to give the two-dimensional structure of **1**. Overlapping ^1^H signals led to several assignment ambiguities in the NOESY spectrum and, thereby, impeded the deduction of the relative configuration. Attempts to crystallize **1** are currently performed in our laboratory.

Following its isolation, **1** was evaluated in an agar diffusion assay against a standard selection of Gram-positive and Gram-negative bacteria as well as some fungi. However, the compound was inactive at all tested concentrations.

A literature search revealed that **1** is the first microbial terpene possessing an octahydro-1*H*-indene backbone, whereas a plant-derived sesquiterpene with this feature was already reported in 1965. Oplopanone (**2**, Figure 3) was originally isolated from *Oplopanax japonicus* [18–19], but can also be found in a number of other plants as well as red algae [20–21]. Comparison of the chemical shifts in **1** with published NMR data for **2** [22] confirmed our assignment of the octahydro-1*H*-indene skeleton.

**Figure 4:**
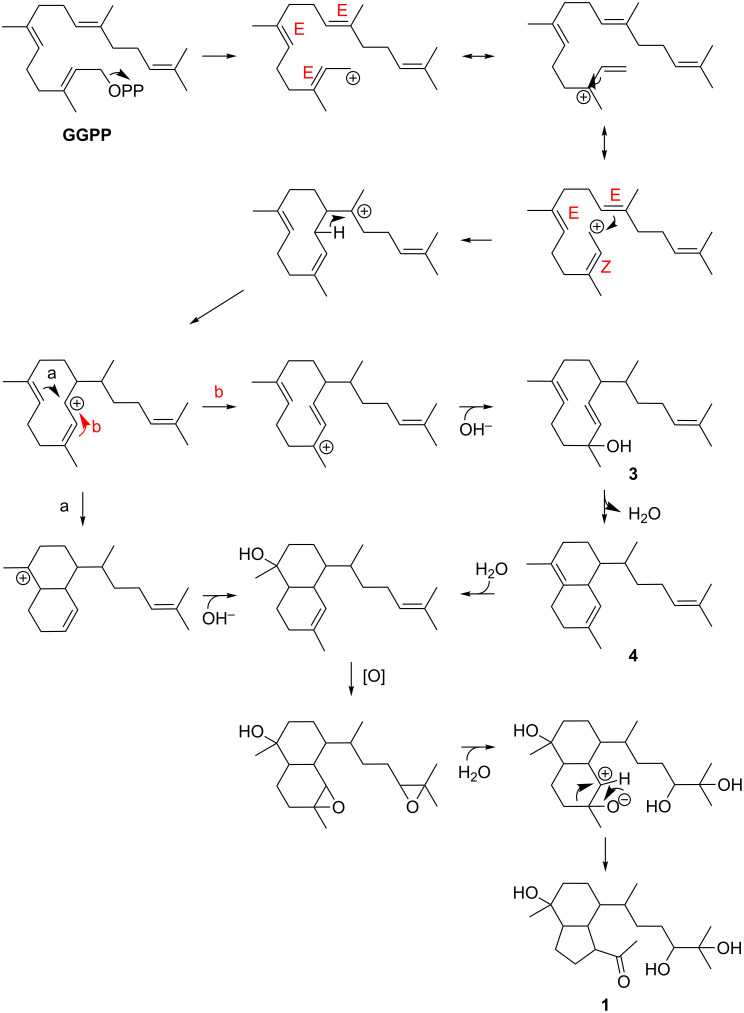
Proposed biosynthesis of **1** via two alternative routes (a) and (b). Route (b) involves the known diterpene obscuronatin (**3**).

The biosynthesis of the bicyclic ring system in **2** was previously proposed to occur via an α-cadinol intermediate, which undergoes a ring contraction reaction [19]. The cadinane family of sesquiterpenes, which also includes α-cadinol, originates from germacrene D [7,23]. In the case of **1**, an analogous pathway can be postulated, which is depicted in Figure 4. The biosynthesis would hence start with geranylgeranyl pyrophosphate (GGPP). Upon ionization, the double bond nearest the diphosphate can adopt a *Z* configuration, thereby facilitating an intramolecular cyclization to a cyclodeca-1,5-diene by electrophilic attack of the allylic carbocation onto the corresponding double bond. A 1,3-hydride shift by Wagner–Meerwein rearrangement followed by another cyclization would then give rise to an octahydronaphthalene cation. Eventually, the addition of OH^−^ would lead to an α-cadinol-type diterpene. For the ring contraction, we would propose a two-step sequence of epoxidation and rearrangement [24], which would directly lead to the acetyl group in **1**. Recently, heterologous expression of the terpene cyclase Haur_2987 in an actinomycete led to a product, which was identified as the soft coral-derived diterpene obscuronatin [25–26]. The biosynthesis of this compound can be easily rationalized via the proposed herpetopanone pathway (route b in Figure 4). Following the formation of the cyclodeca-1,5-diene intermediate, two successive 1,3-hydride shifts and a final addition of OH^−^ would yield obscuronatin (**3**). Interestingly, **3** was observed to undergo facile water elimination to give compound **4** [26]. Obscuronatin might thus serve as a biosynthetic intermediate or, alternatively, as a shunt product in the herpetopanone pathway. An involvement of Haur_2987 in the biosynthesis of **1** seems highly likely.

## Conclusion

In summary, we have identified a new diterpene from the predatory bacterium *H. aurantiacus* 114-95^T^ using an isotope labeling strategy and resolved its structure. A plausible pathway for the biosynthesis of herpetopanone was deduced from its molecular architecture. Furthermore, candidate genes for the necessary ring formations were identified and will be the subject of future investigations.

## Experimental

### General experimental procedures

The IR spectrum was recorded on a benchtop FT-IR 4100 spectrometer (JASCO). HRMS analyses were carried out using an Exactive Mass Spectrometer (Thermo-Scientific). NMR spectra were measured at 300 K on Bruker Avance III spectrometers with chloroform-*d*_1_ (δ_H_ 7.24 ppm; δ_C_ 77.0 ppm) as solvent and internal standard.

### Strain and growth conditions

*H. aurantiacus* 114-95^T^ was obtained from the German Collection of Microorganisms and Cell Cultures (DSMZ). The bacterium was routinely cultured at 30 °C in CY (casitone 3 g/L, yeast extract 1 g/L, CaCl_2_·2 H_2_O 1 g/L, pH 6.8) or VNY liquid medium (sodium glutamate 10 g/L, yeast extract 2 g/L, MgSO_4_·7 H_2_O 2 g/L, NaH_2_PO_4_ 0.10 g/L, Na_2_HPO_4_ 0.05 g/L, pH 6.5) under oxic conditions with gentle shaking (90 rpm).

### Feeding experiments

Feeding experiments were conducted in 2 L Erlenmeyer flasks containing 1 L of VNY liquid medium. The precursors, [1-^13^C]-D-glucose and non-labeled D-glucose, were added aseptically as filter-sterilized aqueous solutions (0.05 M) at the time of inoculation. The fermentation was conducted at 30 °C for 7 days. After centrifugation and removal of the biomass, the supernatant was treated with 10% (w/v) XAD-2 resin (Sigma-Aldrich) in order to adsorb the secreted metabolites. The resin was thoroughly washed with distilled water and, subsequently, eluted with three bed volumes of methanol. The eluates were concentrated under vacuum and the resulting extracts were subjected to LCMS analysis.

### Production and isolation of herpetopanone

The fermentation and extraction conditions were the same as described in the feeding experiments, except that *H. aurantiacus* 114-95^T^ was grown on a 20 L scale in VNY medium and that the cultures were only supplemented with non-labeled D-glucose. The extract that was obtained after the XAD-2 elution was dissolved in 20% (v/v) aqueous MeOH and fractionated by flash column chromatography over Polygoprep C18 (Macherey-Nagel) using an increasing concentration of MeOH in water. Fractions containing **1** were identified by LC–MS analysis, pooled and purified by reversed-phase HPLC on a Shimadzu UFLC liquid chromatography system equipped with a Nucleodur C18 HTec column (VP 250 × 10 mm, 5 μm; Macherey-Nagel) using a gradient from 10% (v/v) MeOH in H_2_O (+ 0.1% trifluoroacetic acid) to 100% (v/v) MeOH over 30 min. The purity of the isolated herpetopanone determined by HPLC was >95% at all wavelengths (compared to sum of total peak areas).

### Agar diffusion assay

The assay was performed as previously described [27]. The test organisms included *Bacillus subtilis* ATCC 6633, *Staphylococcus aureus* SG 511, *Mycobacterium vaccae* IMET 10670, *Escherichia coli* SG 458, *Pseudomonas aeruginosa* K 799/61, *Sporobolomyces salmonicolor* SBUG 549, *Candida albicans* ATCC 14053 and *Penicillium notatum* JP 36.

## Supporting Information

File 1 IR and NMR spectra of herpetopanone. Metabolic profile of *H. aurantiacus* 114-95^T^.
