# Supplementary material for: Herpetopanone, a diterpene from Herpetosiphon aurantiacus discovered by isotope labeling
Source: Beilstein J Org Chem. 2017 Nov 17;13:2458–65. doi: 10.3762/bjoc.13.242 (PMC5704754; doi:10.3762/bjoc.13.242)

Supporting Information

for

Herpetopanone, a diterpene from *Herpetosiphon aurantiacus* discovered by isotope labeling

Xinli Pan[1,2], Nicole Domin[2], Sebastian Schieferdecker[2], Hirokazu Kage[1], Martin Roth[2] and Markus Nett*[1]

Address: [1]Department of Biochemical and Chemical Engineering, Technical Biology, Technical University Dortmund, Emil-Figge-Strasse 66, 44227 Dortmund, Germany and [2]Leibniz Institute for Natural Product Research and Infection Biology, Hans Knöll Institute, Beutenbergstr. 11a, 07745 Jena, Germany

Email: Markus Nett* - markus.nett@bci.tu-dortmund.de

*Corresponding author

IR and NMR spectra of herpetopanone. Metabolic profile of *H. aurantiacus* 114-95^T

Table of contents

Figure S1. Infrared spectrum of herpetopanone SI-3

Figure S2. ^{1}H NMR spectrum of herpetopanone SI-4

Figure S3. ^{1}H-decoupled ^{13}C NMR spectrum of herpetopanone SI-4

Figure S4. DEPT135 spectrum of herpetopanone SI-5

Figure S5. COSY spectrum of herpetopanone SI-5

Figure S6. HSQC spectrum of herpetopanone SI-6

Figure S7. HMBC spectrum of herpetopanone SI-6

Figure S8. NOESY spectrum of herpetopanone SI-7

Figure S9. Metabolic profile of *H. aurantiacus* 114-95^{T} SI-7

Figure S1: Infrared spectrum of herpetopanone.

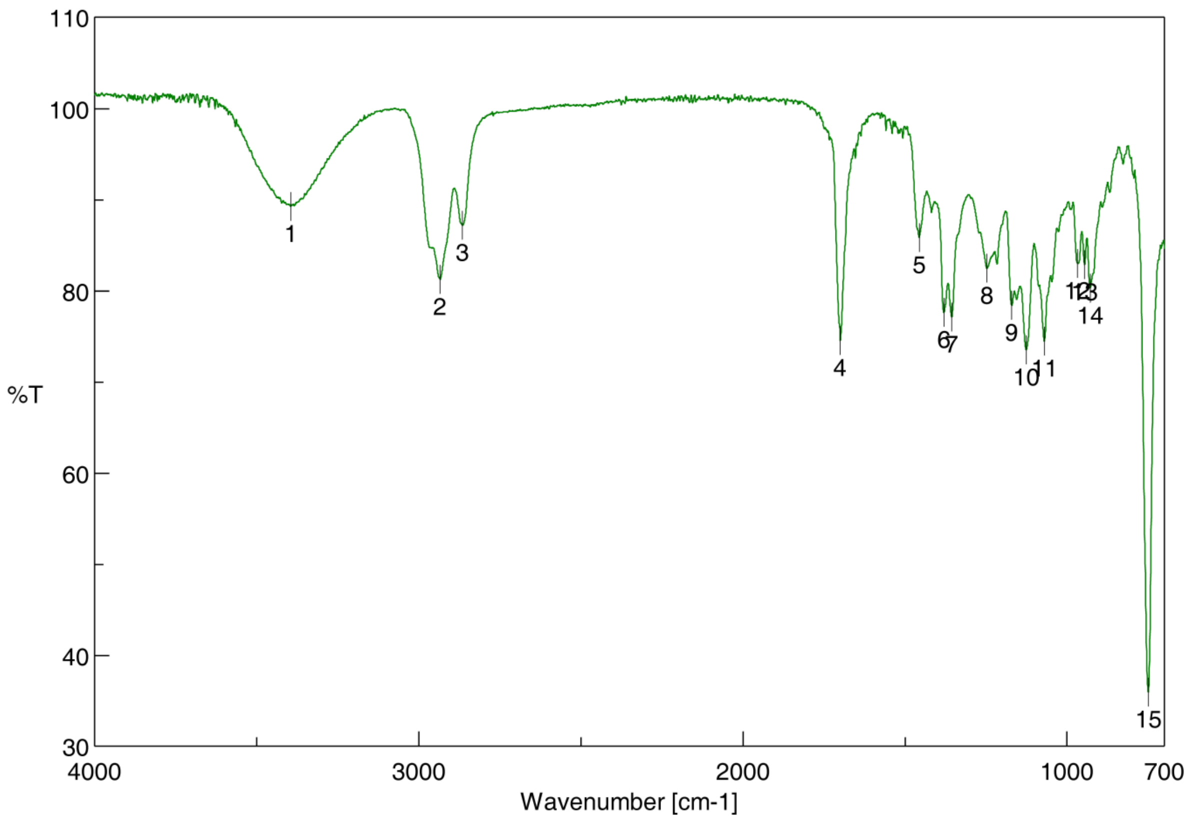

Figure S2: ^{1}H NMR spectrum of herpetopanone in chloroform-d_1.

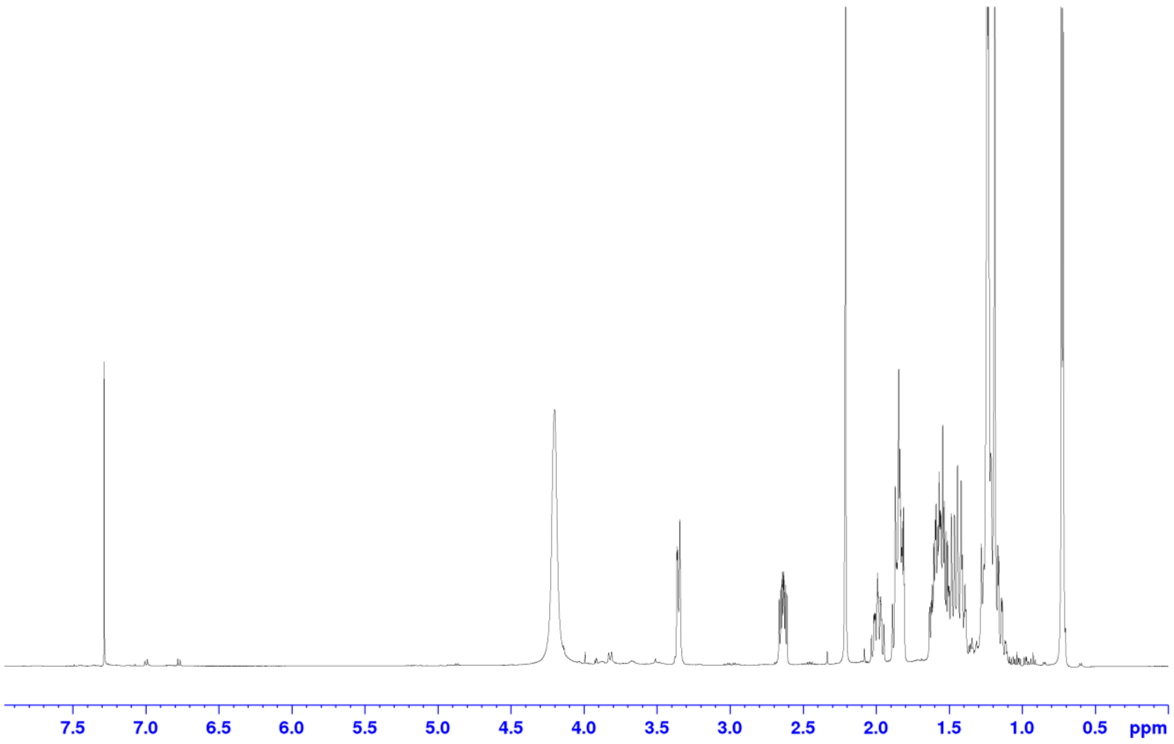

Figure S3: ^{1}H-decoupled ^{13}C NMR spectrum of herpetopanone in chloroform-d_1.

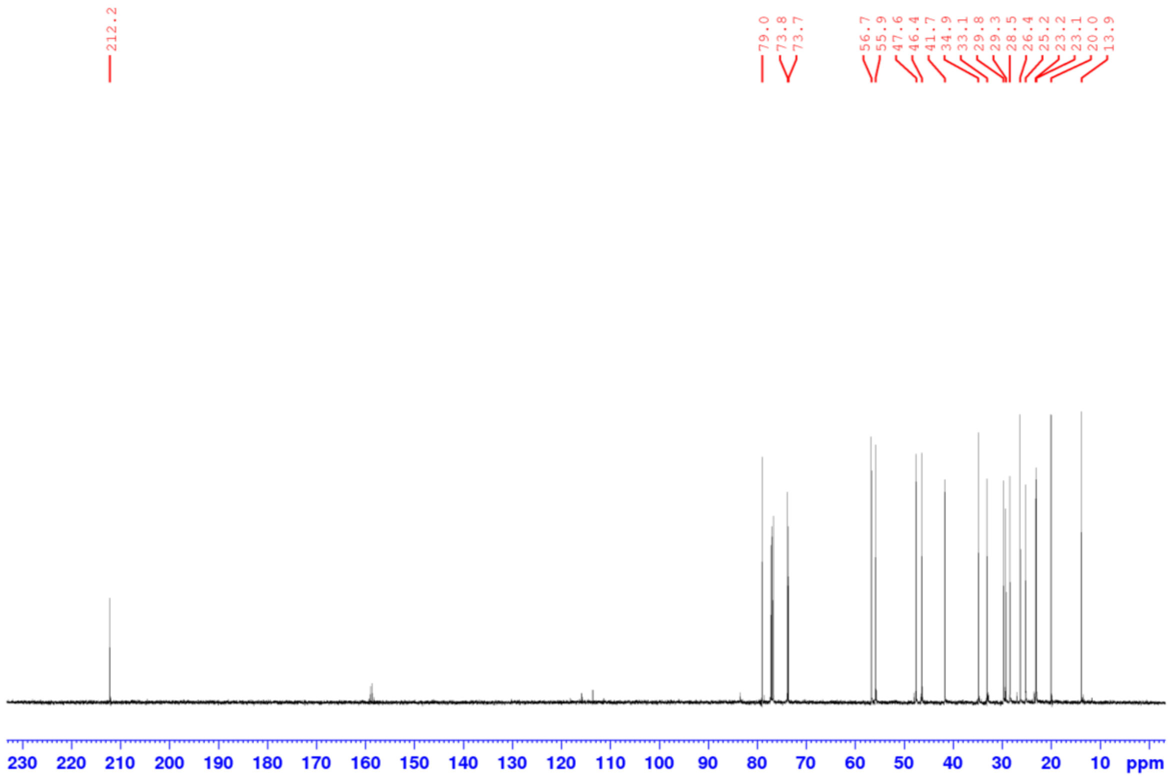

Figure S4: DEPT135 spectrum of herpetopanone in chloroform-d_1.

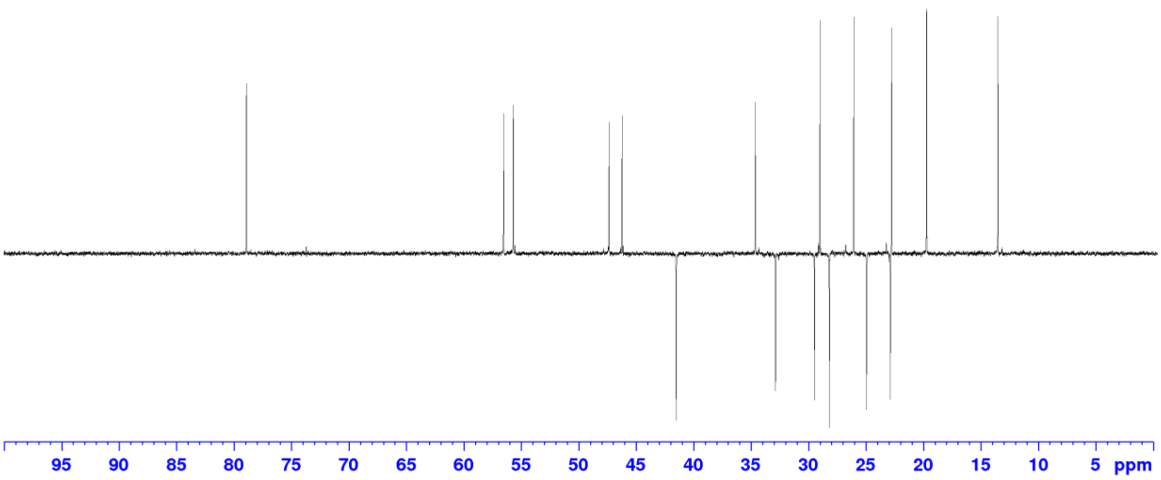

Figure S5: COSY spectrum of herpetopanone in chloroform-d_1.

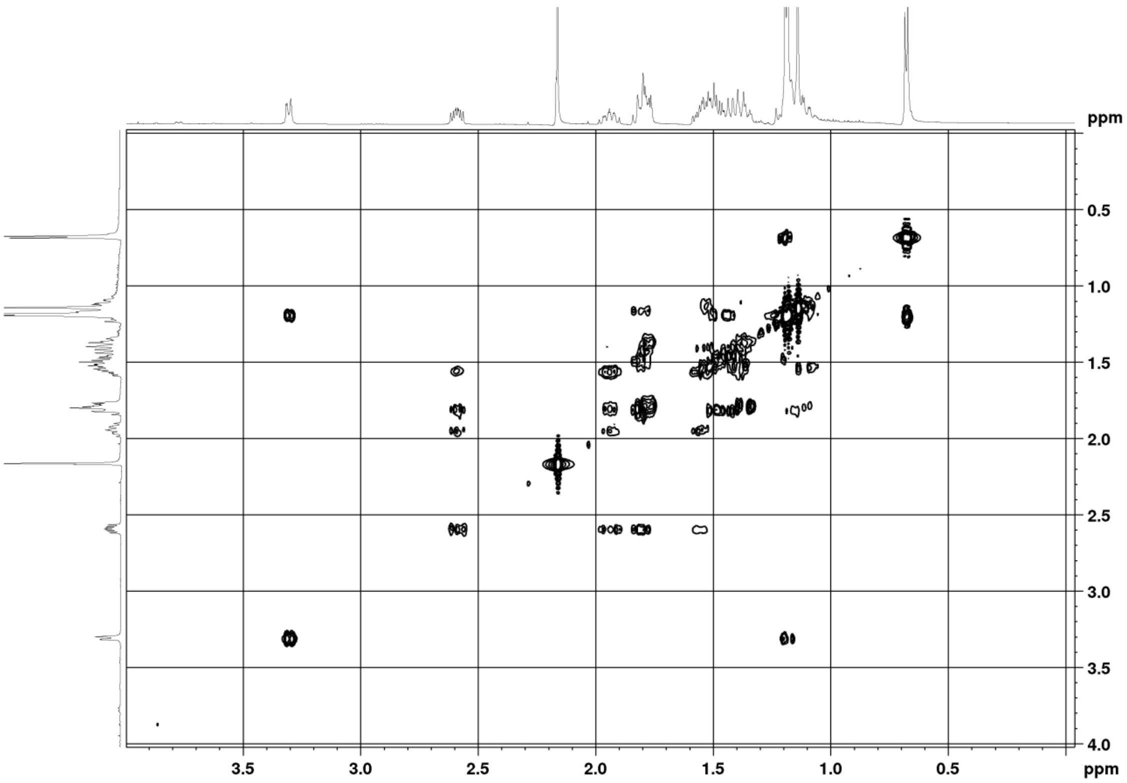

Figure S6: HSQC spectrum of herpetopanone in chloroform-d_1.

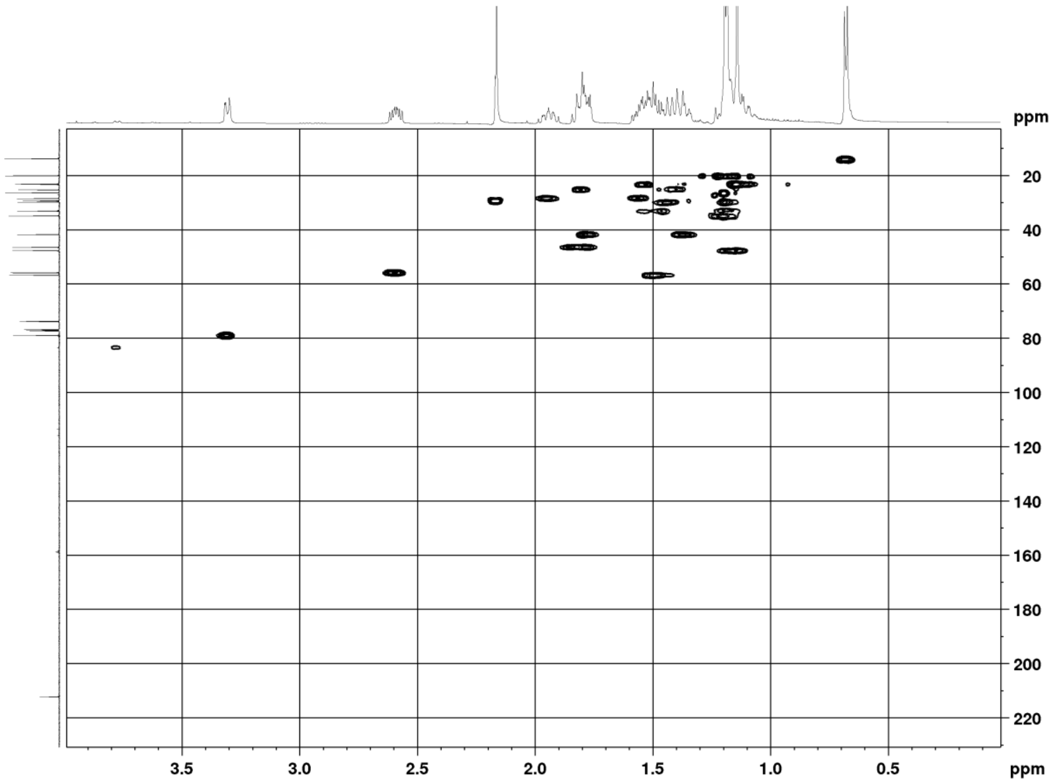

Figure S7: HMBC spectrum of herpetopanone in chloroform-d_1.

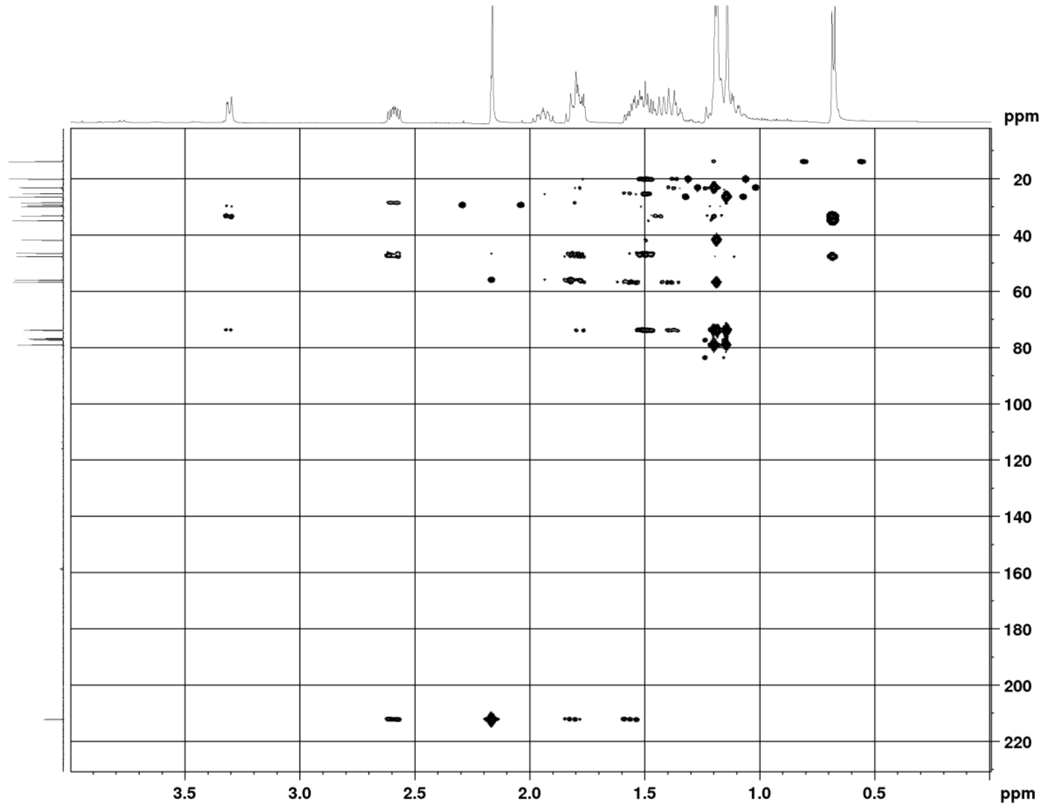

Figure S8: NOESY spectrum of herpetopanone in chloroform-d_1.

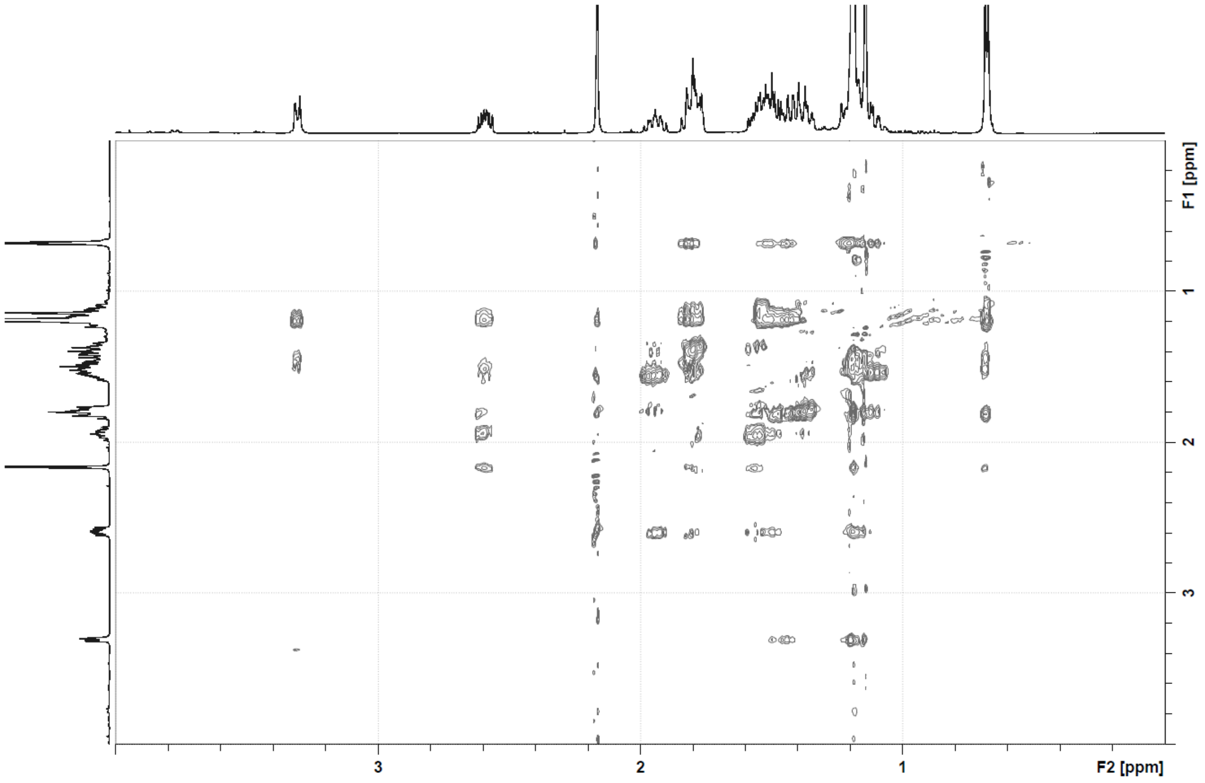

Figure S9: Metabolic profile of *H. aurantiacus* 114-95^T after cultivation in modified VNY medium supplemented with non-labeled D-glucose. Top: Total ion chromatogram (TIC) recorded in positive ion mode. Middle: TIC recorded in negative ion mode. Bottom: UV chromatogram recorded at 254 nm.

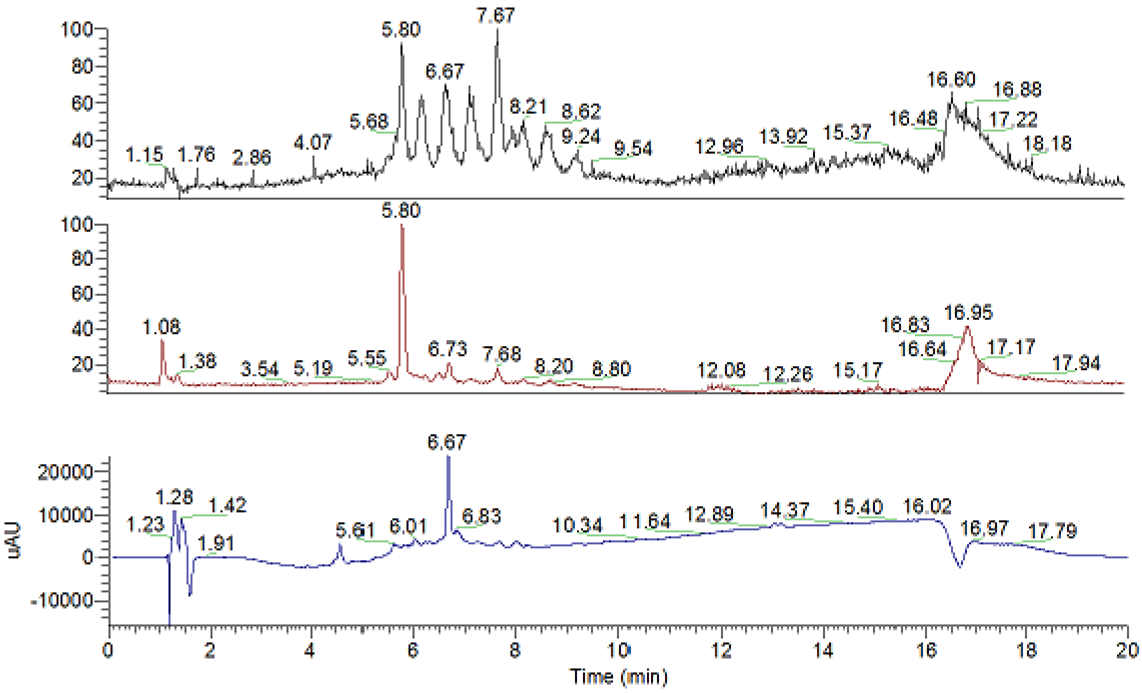

Supplement: File 1 — IR and NMR spectra of herpetopanone. Metabolic profile of H. aurantiacus 114-95T. [file Beilstein_J_Org_Chem-13-2458-s001.pdf]
